# 

**Published:** 1882-08

**Authors:** 


### CONTENTS:

PAGE.

ORIGINAL COMMUNICATIONS:

I. Hereditary Insanity. By A. W. Hagenbach, M.D. .... 113

II. Laryngeal Hemorrhage. By Jefferson Bettman, M.D. ....

NOTES FROM PRIVATE PRACTICE:

III. Syphilitic Cases from Private Practice. By P. O'Connell, M.D. .... 148

IV. Progressive Pernicious Anæmia. By W. Henry, M.D. .... 153

SOCIETY REPORTS:

V. Chicago Medical Society .... 154

VI. Chicago Pathological Society .... 159

VII. Illinois State Board of Health .... 162

VIII. Michigan State Board of Health .... 165

IX. The Tri-State Medical Society .... 169

FOREIGN CORRESPONDENCE:

X. Vienna Letter .... 170

XI. Prag Letter .... 173

DOMESTIC CORRESPONDENCE .... 178

REVIEWS AND BOOK NOTICES .... 181

PAGE.

EDITORIAL:

The New York Code of Ethics .... 196

The Journal of the American Medical Association .... 197

ORIGINAL TRANSLATIONS:

A Visit to the Large Ophthalmological Clinics at Paris .... 199

Action of Arsenic in Diseases of the Skin... 201

Contusion of Popliteal Artery without Damage of the External Parts, followed by Thrombosis and Death .... 203

SELECTIONS:

A Study of the Periods of Incubation of the Communicable Diseases. By B. W. Richardson, M.D. .... 205

Presence of the Micrococcus in the Blood of Malignant Measles; its Importance in Treatment. By John M. Keatiug, M.D... 209

ITEMS .... 147, 153, 169, 180, 195, 198, 204, 223

ANNOUNCEMENTS FOR THE MONTH .... 224

NOTICE TO CORRESPONDENTS .... *advts.*

